# Initial Events in Establishing Vaginal Entry and Infection by Human Immunodeficiency Virus Type-1

**DOI:** 10.1016/j.immuni.2007.01.007

**Published:** 2007-02-23

**Authors:** Florian Hladik, Polachai Sakchalathorn, Lamar Ballweber, Gretchen Lentz, Michael Fialkow, David Eschenbach, M. Juliana McElrath

**Affiliations:** 1 Program in Infectious Diseases, Clinical Research Division, Fred Hutchinson Cancer Research Center, Seattle, WA 98109, USA; 2 Department of Obstetrics and Gynecology, University of Washington School of Medicine, Seattle, WA 98195, USA; 3 Department of Medicine, University of Washington School of Medicine, Seattle, WA 98195, USA; 4 Department of Laboratory Medicine, University of Washington School of Medicine, Seattle, WA 98195, USA; 5 Department of Pathobiology, University of Washington School of Medicine, Seattle, WA 98195, USA

**Keywords:** MICROBIO, CELLIMMUNO, HUMDISEASE

## Abstract

Understanding the initial events in the establishment of vaginal human immunodeficiency virus type-1 (HIV-1) entry and infection has been hampered by the lack of appropriate experimental models. Here, we show in an ex vivo human organ culture system that upon contact in situ, HIV-1 rapidly penetrated both intraepithelial vaginal Langerhans and CD4^+^ T cells. HIV-1 entered CD4^+^ T cells almost exclusively by CD4 and CCR5 receptor-mediated direct fusion, without requiring passage from Langerhans cells, and overt productive infection ensued. By contrast, HIV-1 entered CD1a^+^ Langerhans cells primarily by endocytosis, by means of multiple receptors, and virions persisted intact within the cytoplasm for several days. Our findings shed light on the very earliest steps of mucosal HIV infection in vivo and may guide the design of effective strategies to block local transmission and prevent HIV-1 spread.

## Introduction

The majority of human immunodeficiency type-1 (HIV-1)-infected individuals worldwide are women, and most acquire HIV infection after sexual contact. Blocking HIV mucosal transmission and local spread in the female lower genital tract is therefore key both to prevent infection and ultimately to ease the pandemic. At present, the best strategies to accomplish this are through the design and implementation of efficacious vaccines and topical microbicides that interfere with viral transmission. These efforts require an in-depth understanding of the initial infection events in the mucosa. Previous studies implicated resting and activated CD4^+^ T cells (Gupta et al., 2002; Hu et al., 2004; Veazey et al., 2003; Zhang et al., 1999), while others concluded that Langerhans cells (LC), dendritic cells, and macrophages (Collins et al., 2000; Hu et al., 2000, 2004; Kawamura et al., 2000; Spira et al., 1996) were the likely first targets of HIV-1 infection in the human lower reproductive tract. However, definitive studies of the initial infection in the outer human vaginal epithelium as well as the precise pathways of viral entry into the intraepithelial cells are lacking, which led to our investigation reported here.

Important insights have been gained from HIV infection studies in organ cultures of whole genital mucosa (Collins et al., 2000; Greenhead et al., 2000; Hu et al., 2004; Maher et al., 2005), but some limitations inherent in these models have precluded an explicit analysis of intraepithelial targets. Intraepithelial leukocytes, particularly LC, emigrate from the epithelium shortly after HIV-1 exposure. Thus, observation of intraepithelial infection in situ must occur within the first 24 hr of culture. Alternatively, cells that migrate out of the organ cultures can be examined for HIV-1 infection, but there is no method to determine whether they originated from the mucosal epithelium or the stroma. Lastly, in unidirectional models that allow penetration of HIV from the apical (vaginal) side only, the brick-layered outer epithelial cells prevent most virions from reaching deeper into the epithelium. Consequently, infrequent contact between intact virions and intraepithelial Langerhans and CD4^+^ T cells hinders systematic analysis by microscopy.

To overcome these obstacles, we developed an ex vivo organ culture system that successfully permits separation of the epithelial layer from the vaginal stroma. By exposing isolated epithelial sheets to viable, fluorescence-tagged HIV-1, we can directly evaluate infection of intraepithelial Langerhans and CD4^+^ T cells. In this report, we demonstrated that HIV-1 simultaneously entered both CD1a^+^ Langerhans and CD4^+^ T cells residing in the vaginal epithelium. As evidenced by confocal microscopy in situ, viral entry into intraepithelial CD4^+^ T cells occurred almost exclusively by CD4 and CCR5 receptor-dependent fusion and did not require passage from LC. In contrast, LC efficiently endocytosed HIV-1 and virions survived intact in the cytoplasm for several days. Furthermore, productive infection was readily observed in CD4^+^ T cells but not in LC emigrating from the vaginal epithelium. These findings provide definitive insights into the initial events of HIV-1 infection in the human vagina, which may guide the design of effective strategies to interfere with sexual HIV-1 transmission in vivo.

## Results

### An Ex Vivo Model of Intraepithelial HIV-1 Infection in the Human Vagina

**Figure 1:**
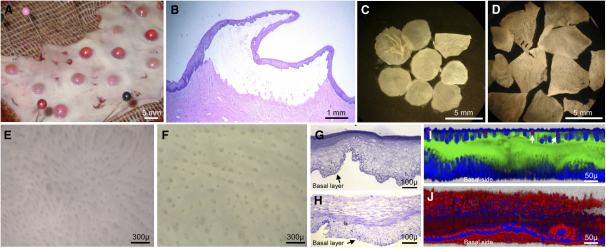
Separation of Vaginal Epithelial Sheets (A) Photograph of suction blisters on surgically excised vaginal mucosa. (B) Hematoxylin-eosin stain of intact suction blister by light microscopy. (C) Blister roofs, i.e., epithelial sheets, floating in PBS after removal from the underlying stroma, visualized under a stereoscope. (D) Epithelial sheets, separated by EDTA treatment. (E) Stereoscopic view of the basal side of a sheet separated by vacuum suction. An organized pattern of rete ridges and depressions, corresponding to the stromal papillae that were pulled out during separation, can be seen. (F) Stereoscopic view of the basal side of an EDTA-separated sheet, exhibiting the same pattern. (G and H) Toluidine blue-stained cross-section of epithelial sheets separated by vacuum suction (G) or ETDA (H), viewed by light microscopy. In the EDTA sheet, the enucleated outer epithelial cells have swollen during the overnight incubation. (I and J) Live/dead cell staining of EDTA-separated sheets. Sheets were stained with calcein AM (live cells, green), ethidium homodimer-1 (nuclei of dead cells, red), and TOPRO-3 (nuclear counterstain, blue) as described in the manufacturer's protocol. Sheets were immersed in glycerol under a coverslip and imaged by confocal microscopy. Stacks covering the complete distance from the basal to the luminal side (toward the vaginal cavity) were acquired, and the sheets were reconstructed in the z-section by Imaris software. (I) In a typical sheet, nearly all cells were alive, staining green, and only few cells at the luminal side were dead, staining red, which represent naturally dying cells that slough off into the vaginal cavity in vivo (two white arrows). (J) Treatment for 1 hr with 1% sodium azide killed all cells, as demonstrated by loss of the green live cell stain and universal acquisition of the red dead cell marker.

To investigate the earliest steps of heterosexual HIV-1 transmission, we developed an ex vivo model that permits direct observation of how HIV-1 targets cells within the outer vaginal epithelium. We separated the squamous vaginal epithelium, the first barrier to the virus, from the underlying stroma, either by 2 hr ex vivo suction blistering or by overnight treatment with 5 mM ethylene diamine tetraacetic acid (EDTA) on ice. Both methods caused gentle disruption along the *lamina lucida* of the basal membrane, which isolated stroma-free epithelial sheets with intact microanatomic structure (Figures 1A–1H) and viability (Figures 1I and 1J). Proteolytic enzymes, which can disrupt surface receptor expression on intraepithelial Langerhans and T cells, were not required. As shown by in situ fluorescence and histochemical staining, expression of the HIV-1 receptors CD4 and CCR5 were retained, as were expression of CD3 on T cells and CD1a, S100, langerin, and HLA-DR on Langerhans cells (see Figures S1 and S2 in the Supplemental Data available online). Within 1 hr of surgical removal of the vaginal mucosa, before suction blistering or EDTA treatment, CCR5 was expressed by the majority of intraepithelial CD3^+^ T cells (83.6% in each of two donors; not shown) and CD1a^+^ LC (53.8%, 63.4%, and 73% in three donors; Figure S1A). The majority of intraepithelial T cells bore the CD45RO^+^ memory phenotype (Figure S3). After suction blistering, 71.6% (mean, range 49.7%–97.0%) of CD3^+^ T cells expressed CCR5 in cell suspensions generated by mechanical dispersion of epithelial sheets in three tissue donors (not shown). Of CD3^+^ T cells isolated from EDTA-separated sheets in three additional donors, 78.9% (mean, range 56.1%–95.1%) expressed CCR5, and 27.3% (mean, range 12.6%–54.4%) expressed CD4 (Figure S1B). Because the cell suspensions contained too few individual CD1a^+^ LC for flow cytometric evaluation, we analyzed CCR5 and CD4 expression on LC by labeling in situ. In EDTA-separated sheets, 44.5% (mean, range 32.2%–56.3%; n = 4 donors) of vaginal LC expressed CCR5, and 54.3% (mean, range 36.8%–83.7%; n = 3 donors) expressed CD4 (Figures S1C and S1D). Isotype control staining of the LC revealed an average nonspecific background in 3.7%. These findings indicated that both intraepithelial Langerhans and T cells in the human vagina expressed the receptor for CCR5-tropic HIV-1 infection. Moreover, CCR5 expression persisted throughout the period necessary to obtain the epithelial sheets by suction blistering or EDTA treatment. Thus, the experimental procedures were suitable to test the susceptibility of these cells to HIV-1 infection.

### Confocal Microscopy Studies of HIV-1 Binding and Entry In Situ

**Figure 2:**
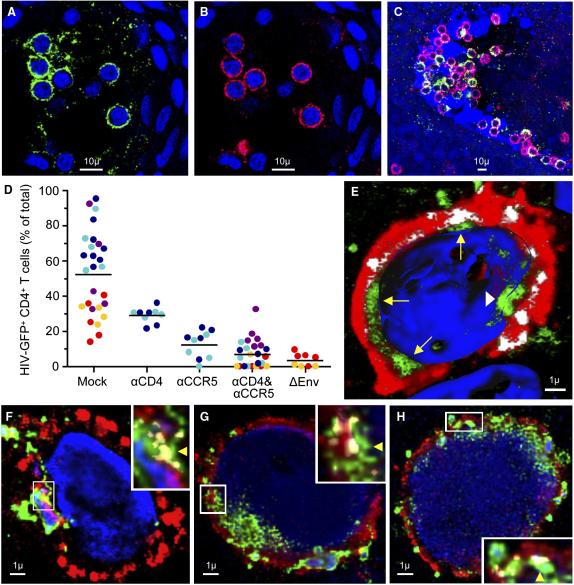
Binding and Entry of HIV-1 in Intraepithelial Vaginal T Cells Suction blister sheets were spinoculated for 2 hr with GFP-Vpr-tagged HIV-1_JR-CSF_, stained for cell-specific markers and analyzed by confocal microscopy. GFP^+^ virions are shown in green and CD4 in red. Yellow (or white in [E]) signifies coexpression of GFP and CD4. The blue nuclear counterstain is TOPRO-3. (A–C) Clusters of virion binding CD4^+^ T cells in the vaginal epithelium in two donors ([A] and [B], donor 1; [C], donor 2). (D) Blocking of viral binding with antibodies to CD4 (αCD4) or CCR5 (αCCR5). Viral binding was quantified with an algorithm given in Supplemental Experimental Procedures online. Each dot depicts the percent GFP^+^ T cells among all CD4^+^ T cells counted in a distinct, nonoverlapping confocal stack. Each color signifies stacks acquired in the same tissue donor. Horizontal black bars represent the means calculated from the average percentages in each donor. Mock versus αCD4 and αCCR5 blocking (p = 0.007) was evaluated for significance as described in Experimental Procedures. ΔEnv HIV-1 lacks the viral envelope. (E) Three-dimensional reconstruction from a confocal image stack of an intraepithelial CD4^+^ T cell by Imaris software. The cell was virtually clipped at its widest circumference so that the green virions located inside the cytoplasm, between the red cell membrane and the blue nucleus, can be clearly identified (yellow arrows). Areas where HIV-1 penetrates the cell membrane, signified by CD4 and HIV-1 colocalization, are shown in white color. The nucleus is rendered as an isosurface and virions appear to enter it at one location (white arrowhead). (F–H) Three representative CD4^+^ T cells exhibiting cytoplasmic entry of virions. Confocal stacks of individual cells were deconvolved with Autodeblur, and viral entry was determined with an algorithm described in Supplemental Experimental Procedures online. The yellow arrowheads in the magnified insets point to areas of viral entry where GFP^+^ virions are located on the cytoplasmic side along a section of the cell membrane costaining for CD4 and GFP.

To evaluate initial HIV binding and entry in intraepithelial Langerhans and T cells, we performed in situ studies with the epithelial sheets and fluorescent-labeled HIV-1 virions. Suction blister sheets from five donors were challenged with infectious CCR5-tropic HIV-1 JR-CSF tagged by incorporation of green fluorescent protein (GFP) fused with the viral protein R (Vpr) (McDonald et al., 2003), stained for cell-phenotypic markers, visualized by confocal microscopy, and analyzed quantitatively. Many intraepithelial CD4^+^ T cells bound the green fluorescent virions (Figures 2A–2C). For these five donors, 49.7% (mean, range 26.5%–69.9%) of CD4^+^ T cells (≥100 CD4^+^ cells/donor were counted) exhibited viral binding (Figure 2D). Preincubation with a cocktail of CCR5 and CD4 monoclonal antibodies (mAb) decreased viral binding to 6.4% (mean, range 1.4%–17.4%) of CD4^+^ T cells (p = 0.007) (Figure 2D). Blocking with anti-CCR5 alone in tissue from two of five donors was nearly as effective (from 69.9% and 68.0% without blocking, to 9.1% and 15.7%, respectively, with blocking), whereas anti-CD4 alone in the same two donors blocked with lower efficiency (to 28.3% and 29.6%, respectively) (Figure 2D). In another two donors, we also tested a GFP-Vpr-tagged envelope-deficient virus, which exhibited fluorescence comparable to the complete virus when spotted on glass slides (not shown), but bound only to 0.4% and 5.3% of CD4^+^ T cells in situ (versus 29.8% and 26.5% with complete virus) (Figure 2D).

To determine whether HIV-1 binding to intraepithelial CD4^+^ T cells led to cytoplasmic entry of virions, we acquired image stacks of 25 randomly selected HIV^+^ cells in each of four donors (60× magnification) and performed colocalization studies of GFP-Vpr-tagged virions with the CD4 label of the cell membrane (Figures 2E–2H). To distinguish virions within the cytoplasm from those aligned extracellularly to invaginated cell membrane, entry was determined on the slice of the image stack displaying the widest T cell circumference, as shown in Figure 2E and Movie S1. The presence of a green GFP signal on the cytoplasmic side, adjacent to an area of the cell membrane where the red CD4 and the green GFP signal colocalized (yellow in Figures 2F–2H), signified viral entry, which was observed in 74% (mean, range 48%–88%) of the analyzed HIV^+^ T cells.

**Figure 3:**
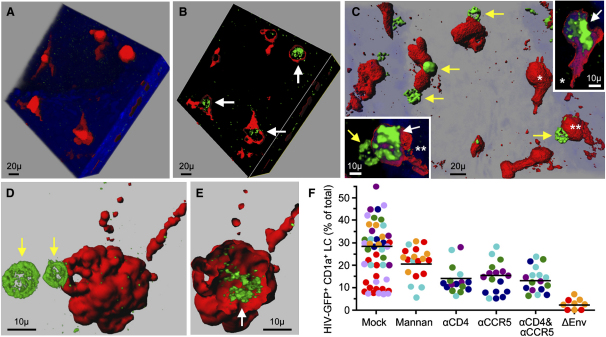
Entry of HIV-1 into Intraepithelial Vaginal Langerhans Cells Suction blister sheets were spinoculated for 2 hr with GFP-Vpr-tagged HIV-1_JR-CSF_, stained for cell-specific markers, and analyzed by confocal microscopy. GFP^+^ virions are shown in green, CD1a in red. The blue nuclear counterstain TOPRO-3 is rendered more or less transparent. Confocal stacks were recreated in three dimensions with Imaris software. (A–E) Representative virion-containing LC in three different donors ([A] and [B], donor 1; [C], donor 2; [D] and [E], donor 3). x, y, and z planes are rotated in (A) and (B) to illustrate how a virtual clipping plane can be sliced through the tissue to visualize cross-sections. White arrows point to cross-sections of LC containing intracellular HIV-1. Yellow arrows point to virus-laden T cells conjugated to LC. (F) Blocking of viral entry with mannan, CD4 (αCD4), or CCR5 (αCCR5) antibody. Viral entry was quantified with an algorithm given in Supplemental Experimental Procedures online. Each dot depicts the percent GFP^+^ LC among all CD1a^+^ LC counted in a distinct, nonoverlapping confocal stack. Each color signifies stacks that were acquired in the same tissue donor. Horizontal black bars represent the means calculated from the average percentages in each donor. Mock versus mannan blocking (p = 0.54) and mock versus αCD4 and αCCR5 blocking (p = 0.008) were evaluated for significance as described in Experimental Procedures.

In LC, the ratio of cytoplasm to nucleus is usually much larger than in T cells, and cytoplasmic entry of GFP-Vpr-tagged virions was therefore easily discerned without formal colocalization studies. CD1a^+^ LC accumulated around the depressions formed by the stromal vascular papillae, and many contained GFP-Vpr-tagged HIV-1_JR-CSF_ virions after 2 hr of viral challenge (Figures 3A–3E). Rather than binding to the cell surface, HIV readily entered the cytoplasm, indicating that viral internalization occurred very rapidly. Frequently, LC clustered with virus-laden lymphocytes (Figures 3C and 3D). In seven donors (≥65 LC/donor were counted), 28.6% (mean, range 15.0%–37.8%) of LC exhibited viral entry (Figure 3F). In four donors, viral entry in 32.8% of LC (mean, range 27.2%–37.8%) was reduced to 15.6% (mean, range 8.1%–19.7%; p = 0.024) with CCR5 mAb, 14.2% (mean, range 11.1%–17.3%; p = 0.006) with CD4 mAb, and 13.3% (mean, range 12.1%–15.9%; p = 0.008) with both CCR5 and CD4 mAbs (Figure 3F). Preincubation of epithelial sheets with 100 μg/ml mannan, which inhibits binding of HIV-1 to C-type lectin receptors (Geijtenbeek et al., 2000), decreased viral internalization only marginally in three donors tested, from 25.3% (mean, range 15.0%–33.6%) in mock-treated sheets to 20.7% (mean, range 15.5%–23.8%; p = 0.54) (Figure 3F). Envelope-deficient virus, tested in two donors, exhibited residual internalization in 3.4% and 1.5% of LC (Figure 3F).

Taken together, our in situ studies demonstrated that CCR5-tropic HIV-1 efficiently entered both CD4^+^ T cells and CD1a^+^ LC residing in the vaginal epithelium. In CD4^+^ T cells, HIV-1 entry occurred almost exclusively via the CD4 and CCR5 receptors. In LC, viral entry was partially dependent on CD4 and CCR5, whereas C-type lectin receptors appeared to play a more marginal role.

### Electron Microscopy of HIV-1 Binding and Entry In Situ

**Figure 4:**
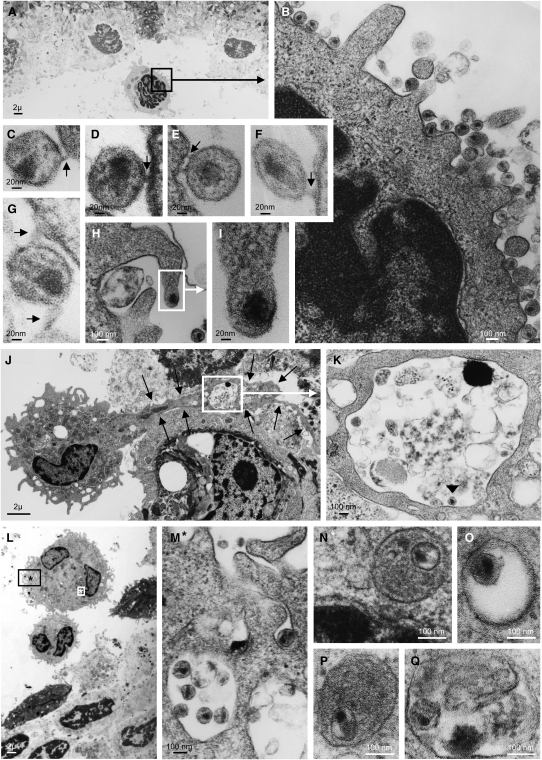
Electron Microscopy of Viral Interaction with Intraepithelial Lymphocytes and Langerhans Cells Epithelial sheets were spinoculated with HIV-1_BaL_ for 2 hr and processed for electron microscopy. (A–G) Representative images of viral binding to intraepithelial lymphocytes. Black arrows point to bridges forming between viral envelope and cell membrane. The region within the black rectangle in (A) is magnified in (B). (C–G) Images magnified from additional lymphocytes. Cytoplasmic entry of intact, enveloped virions into lymphocytes was not seen. (H and I) A possible HIV-cell membrane fusion event in another lymphocyte. The region within the white rectangle in (H) is magnified in (I). (J and K) Emigrating HIV-1 containing LC in one donor, displaying a long cytoplasmic process retained in the epithelium (black arrows). The region within the white rectangle in (J) is magnified in (K) and demonstrates that the LC process contains a vacuole with one virion inside (black arrowhead). (L–N) HIV-1-containing LC in a different donor. (L) Overview. (M) Cell region indicated by the black rectangle and asterisk in (L), displaying surface-bound virions. (N) Cell region indicated by the white rectangle and asterisk in (L), displaying an endosomal vesicle located adjacent to the cell nucleus and containing one intact virion. (O–Q) Endosomal vesicles found deep inside the cytoplasm, containing intact virions in three additional LC.

GFP-Vpr-tagged viral particles found inside the cytoplasm of a cell may represent either sequestered intact virions or viral core complexes released into the cytoplasm after fusion of the viral envelope with the cell membrane. To determine whether intraepithelial Langerhans and T cells internalize intact virions, we challenged vaginal epithelial sheets from three donors with high-dose CCR5-tropic HIV-1_BaL_, and we then examined the tissue for virus by electron microscopy. In all three donors, we found intraepithelial lymphocytes with virions attached to their surface, sometimes in large numbers, with thin bridges forming between the viral envelope and the cell membrane (Figures 4A–4G). Some events were indicative of fusion (Figures 4H and 4I). In one donor, we systematically counted 200 intraepithelial lymphocytes and determined that 26% of these had at least one virion attached to their cell membrane. Despite this high rate of viral binding, intact virions were never found inside intraepithelial lymphocytes in any of the three donors. In contrast, intact virions were present within the cytoplasm of vaginal LC in all three donors, often within endosomal compartments located adjacent to the nucleus (Figures 4J–4Q). In one donor, we systematically counted 160 LC, of which 18.7% contained intact cytoplasmic virions. Thus, endocytosis of intact virions was common in intraepithelial vaginal LC but not in lymphocytes.

### Productive HIV-1 Infection of Langerhans versus CD4^+^ T Cells in the Vaginal Epithelium

**Figure 5:**
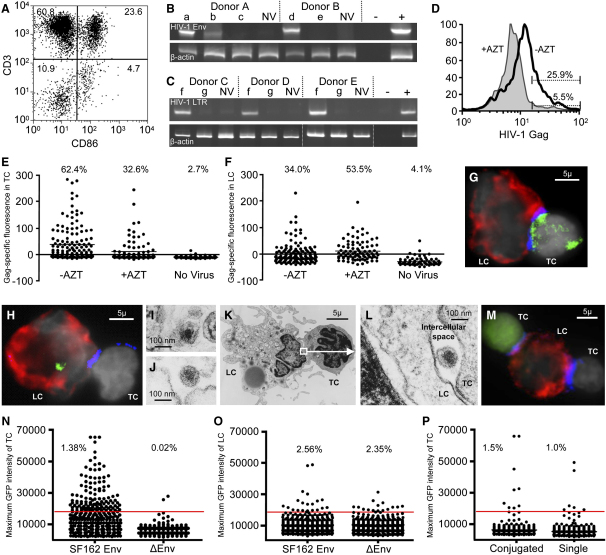
Productive HIV-1 Infection in Langerhans and T Cells Emigrating from the Vaginal Epithelium (A) FACS analysis of cells emigrating from an EDTA-separated sheet. Most cells were single T cells (upper left quadrant) and LC-TC conjugates (upper right quadrant). Only few single LC were seen (lower right quadrant). Contaminating epithelial cells are located in the lower left quadrant. Percentages of cells in each quadrant are indicated. (B) Proviral PCR assay for HIV-1 *env* DNA in cells emigrating from virus-exposed vaginal epithelial sheets separated by vacuum suction in two donors. (C) Alu-LTR PCR assay for integrated HIV-1 DNA in cells emigrating from virus-exposed sheets separated by EDTA in three donors. (B and C) Epithelial sheets were placed for 1 hr in Hanks buffered salt solution containing 5 mM calcium chloride to reverse the reported (Dimitrov et al., 1993; Klimstra et al., 2003) inhibitory effect of calcium depletion on HIV infectivity. Sheets were then exposed to HIV-1 in the presence or absence of AZT for 2 hr, washed vigorously, and cultured in the presence or absence of AZT for 48 hr. Emigrated LC and T cells were harvested and DNA was isolated and subjected to the PCR assays. Abbreviations: a, BaL; b, SF-2; c, BaL + AZT; d, BaL + SF-2; e, BaL + SF-2 + AZT; f, JR-CSF; g, JR-CSF + AZT; NV, no virus; −, no template control; +, infected PHA blasts. (D–H) HIV-1 Gag expression. Vaginal epithelial sheets were exposed to HIV-1_JR-CSF_ in the presence or absence of AZT for 2 hr and washed vigorously. Sheets were then cultured in the presence or absence of AZT for 60 hr, and emigrated LC and T cells were harvested, stained for HIV-1 Gag and cell-specific markers, and analyzed by FACS or fluorescence microscopy. (D) FACS analysis in one tissue donor representative of two. 7-AAD^−^ live CD3^+^ T cells were gated, and the expression of HIV-1 Gag after HIV-1 (−AZT) or HIV-1 + AZT (+AZT) exposure are overlaid as two histograms. The percentages of positive cells, as determined by comparison to IgG isotype staining, are indicated. (E–H) Fluorescence microscopy analysis in one tissue donor representative of three. Gag-specific fluorescence for T cells (E) and LC (F) within LC-TC conjugates was determined as described in Supplemental Experimental Procedures online. Percentages of positive cells are indicated. (G) Example of a Gag^+^ T cell in an emigrated LC-TC conjugate. Gag expression is shown in green, HLA-DR in red, CD3 in blue, and the nuclear counterstain in gray. (H) Example of a Gag^+^ LC in an emigrated LC-TC conjugate. (I–L) Electron microscopy of emigrated LC-TC conjugates. Epithelial sheets were spinoculated with HIV-1_BaL_ for 2 hr and cultured for 60 hr. Emigrated LC-TC conjugates were harvested and processed for electron microscopy. (I and J) HIV-1 virions located within the cytoplasm of two emigrated LC. (K and L) HIV-1 virion located in the intercellular space along the contact zone between a T cell and a LC. (M–P) GFP expression from GFP-encoding HIV-1. Vaginal epithelial sheets were exposed for 2 hr to GFP-encoding, single round infection-competent HIV-1, either enveloped with CCR5-tropic SF162 Env (SF162 Env) or Env-deficient (ΔEnv). Sheets were cultured for 60 hr and emigrated LC and T cells were harvested, stained for cell-specific markers, and analyzed by fluorescence microscopy. Example of a GFP^+^ T cell within an emigrated LC-TC conjugate (M). GFP expression is shown in green, HLA-DR in red, CD3 in blue, and the nuclear counterstain in gray. Maximum GFP intensity for T cells (8258 cells for SF162 Env and 9729 cells for ΔEnv) (N) and LC (819 cells for SF162 Env and 767 cells for ΔEnv) (O) was determined as described in the Supplemental Experimental Procedures online. (P) Comparison of maximum GFP intensity for T cells conjugated to LC versus unconjugated single T cells. In the same sample as in (N), conjugated and unconjugated T cells were categorized by visual inspection on the computer screen and their maximum GFP intensities plotted. A total of 616 conjugated T cells were present in the sample and compared to an equal number of unconjugated T cells. The red lines in (N)–(P) represent an arbitrary positive-negative cut-off. Percentages of positive cells are indicated. Results shown are for one representative donor out of two.

To determine whether HIV-1 binding and entry led to productive infection, we challenged epithelial blister sheets from two donors with HIV-1_BaL_ and EDTA sheets from three additional donors with HIV-1_JR-CSF_. After culturing the HIV-exposed sheets for 60 hr, we harvested the cells that migrated from tissue into the culture medium. The emigrated cells consisted of single HLA-DR^+^ Langerhans and CD3^+^ T cells, Langerhans cell-T cell (LC-TC) conjugates, and contaminating epithelial cells (Figure 5A). Single LC were much scarcer than either LC-TC conjugates or single T cells (4.7% in Figure 5A), which was consistent with our prior experience when we used whole cervicovaginal mucosa (Hladik et al., 1999a). We then analyzed the cells for HIV-1 DNA provirus (in blister sheets) and specifically integrated viral DNA (in EDTA sheets). Proviral and integrated HIV-1 DNA were clearly present in emigrated cells from virus-exposed epithelial sheets, whereas no viral DNA was detectable when the tissue was exposed to HIV-1 in the presence of azidothymidine (AZT) or to mock virus (Figures 5B and 5C). These findings demonstrated that CCR5-tropic HIV-1 initiated productive infection in cells that could eventually migrate out of vaginal epithelial sheets.

Because the emigrated cells contained too few unconjugated LCs for further purification by fluorescence-activated cell sorting (FACS), we did not specifically identify the infected cell type(s) in these PCR assays. Instead, to delineate whether intraepithelial CD4^+^ T cells, LC, or both are productively infected, we evaluated viral protein production by flow cytometry (T cells) and fluorescence microscopy (T cells and LC). Because of the migratory properties of intraepithelial T cells and LC (Figure 5A), de novo protein production in these cells could not be measured conveniently in situ. We therefore challenged epithelial sheets with HIV-1_JR-CSF_ with or without 10 μM AZT, harvested the emigrated T cells and LC after 60 hr, and then stained the cells for expression of CD3, HLA-DR, and HIV-1 Gag.

By flow cytometry, we determined in two HIV-1-exposed samples that 25.9% and 15.5% of emigrated, unconjugated CD3^+^ T cells expressed HIV-1 Gag compared to 5.5% and 3.5%, respectively, in the HIV-1 + AZT controls (Figure 5D). Emigrated cells from three additional donors were examined by fluorescence microscopy. In these, 62.4%, 20.0%, and 7.3% of the conjugated T cells exposed to HIV-1 were Gag positive, compared to 32.6%, 7.7%, and 3.3%, respectively, in the HIV-1 exposed, AZT-treated controls (Figure 5E) (p = 0.024 for microscopy and FACS samples combined). In the same LC-TC conjugates, 40.0%, 34.0%, and 5.5% of the LC were Gag positive in the HIV-1-exposed samples, compared to 7.7%, 53.5%, and 4.2%, respectively, in the HIV-1 + AZT controls (Figure 5F) (p = 0.784). Mock infection in two of these donors revealed nonspecific background in 3.7% of the conjugated T cells and 3.2% of the LC. Representative examples of a Gag-positive T cell and a Gag-positive LC within LC-TC conjugates are shown in Figures 5G and 5H. These findings demonstrated de novo Gag protein synthesis and thus productive CCR5-tropic HIV-1 infection in T cells emigrating from the vaginal epithelium. In contrast, comparable amounts of HIV-1 Gag staining in LC infected with HIV-1 with or without AZT treatment suggested that residual internalized input virions obscured any de novo protein production that may have occurred.

To determine whether virions that LC endocytosed survived intact within the cytoplasm, we scanned emigrated LC in one donor by electron microscopy. 3 days after HIV-1_BaL_ challenge of the vaginal epithelium, emigrated LC still carried intact cytoplasmic virions (Figures 5I and 5J). Intact HIV-1 was also noted in the intercellular space of the LC-TC contact zone (Figures 5K and 5L). These observations suggested that endocytosed HIV-1 virions persisted in LC during emigration and thus remained accessible for in trans infection of susceptible neighboring CD4^+^ T cells.

To further ascertain whether vaginal epithelial T cells and LC supported productive infection with CCR5-tropic HIV-1, we challenged epithelial sheets from two donors with GFP-encoding, single round infection-competent HIV-1 containing SF162 Env. After harvesting the emigrated cells after 60 hr of culture, we stained for CD3 and HLA-DR and determined GFP expression by fluorescence microscopy. Because the intact virions themselves were not fluorescent and Gag labeling was not required, residual input virus was not detected in this assay. This approach confirmed productive infection of T cells emigrating from HIV-1-exposed vaginal epithelium. A representative GFP-positive T cell within a LC-TC conjugate is shown in Figure 5M. GFP expression was present in 1.38% and 0.36% of HIV-1_SF162_-exposed CD3^+^ T cells, in contrast to 0.02% and 0.08%, respectively, of CD3^+^ T cells exposed to GFP-encoding envelope-deficient HIV-1 (Figure 5N). However, we were unable to detect productive infection of HLA-DR^+^ LC with this method. In the HIV-1_SF162_-exposed samples, 2.56% and 2.17% of LC were GFP positive, compared to 2.35% and 2.62%, respectively, in the ΔEnv control samples (Figure 5O). These findings confirmed productive infection in intraepithelial vaginal CD4^+^ T cells, whereas productive infection in Langerhans cells was either inefficient or absent.

To determine whether LC enhanced productive T cell infection, we compared the percentage of productively infected, emigrating T cells when conjugated or unconjugated with LC. The ratios of productive infection found in conjugated versus unconjugated T cells was 1.44 and 1.52 in the two tissue samples infected with the GFP-encoding, single round infection-competent HIV-1_SF162_ (Figure 5P). Also, the ratio of Gag-positive conjugated compared to unconjugated T cells in one sample exposed to HIV-1_JR-CSF_ was 1.16. Thus, productive HIV-1 infection of T cells was not dependent on stable conjugation to LC, although LC appeared to exhibit a slightly enhancing effect.

### Distribution of HIV in LC-TC Conjugates of the Vaginal Epithelium

**Figure 6:**
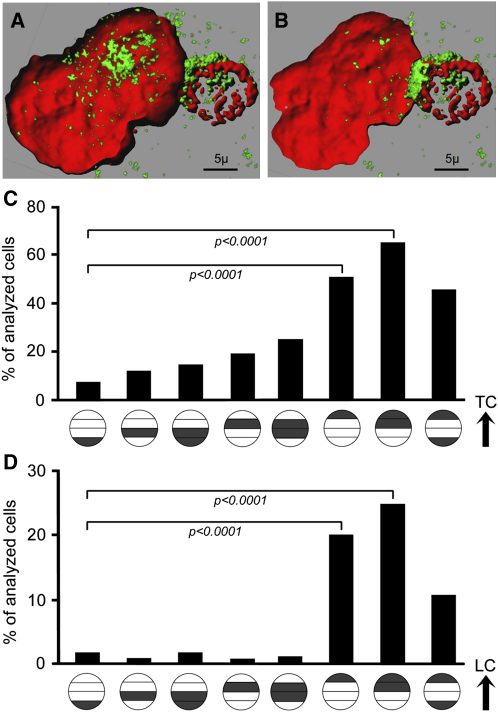
Recruitment of HIV-1 toward the LC-TC Contact Zone (A and B) GFP^+^ virion distribution in a LC-TC conjugate in situ. Suction blister sheets were spinoculated for 2 hr with GFP-Vpr-tagged HIV-1_JR-CSF_, stained for cell-specific markers, and analyzed by confocal microscopy. GFP^+^ virions are shown in green, HLA-DR in red. The blue nuclear counterstain TOPRO-3 is rendered transparent. Confocal stacks were recreated in three dimensions with Imaris software. (C and D) HIV-1 Gag distribution in LC-TC conjugates after emigration. Vaginal epithelial sheets from four donors were exposed to HIV-1_JR-CSF_ for 2 hr, washed vigorously, and cultured for 60 hr. Emigrated LC and T cells were harvested, stained for HIV-1 Gag and cell-specific markers, and analyzed by fluorescence microscopy. The cellular distribution of HIV-1 Gag was categorized and quantified with Metamorph 6.2. Each HIV-1 Gag^+^ cell within a LC-T cell conjugate was divided into four segments laid out in parallel to the LC-T cell junction, and the fraction of the total green fluorescence signal in each segment was determined. (C) Distribution of HIV Gag in conjugated LC. Each of the circles along the x axis categorizes a specific HIV Gag distribution pattern within the LC. A single dark gray band indicates that at least 50% of the total green fluorescence was found within this segment of the LC. Two dark gray bands indicate that at least 75% of the total green fluorescence was found within these two segments of the LC. The black arrow indicates the orientation of the LC in relationship to the contact zone with the T cell. An individual cell could be assigned to more than one category. As an example, the distribution of HIV Gag in the LC depicted in Figure 5H places the cell into the second, fourth, and fifth categories from the right. p values were determined by two-tailed chi-square test. (D) Distribution of HIV Gag in conjugated T cells. The HIV Gag distribution pattern within each conjugated T cell was categorized as described in (C). The black arrow indicates the orientation of the T cell in relationship to the contact zone with the LC. As an example, the distribution of HIV Gag in the T cell depicted in Figure 5G places the cell into to the first category from the right. p values were determined by two-tailed chi-square test.

Recruitment of HIV-1 to an “infectious synapse” between dendritic cells and T cells has been reported (McDonald et al., 2003), a formation that may facilitate the passage of virions between the two cell types and enhance viral infectivity. Because we frequently observed LC-TC conjugates in situ as well as after emigration from the vaginal epithelium (Figure 5A), we asked whether infectious synapse formation did indeed occur between these intraepithelial LC and T cells. We challenged vaginal epithelial sheets from two donors with GFP-Vpr-tagged HIV-1_JR-CSF_ for 2 hr. After fluorescence staining of the sheets for cell-specific markers, we scanned 30 GFP^+^ LC-TC conjugates in each donor at 60× for enrichment of bound or internalized virus toward the Langerhans-T cell junction. Although virions concentrated at the synapse in a few conjugates (Figures 6A and 6B), the viral distribution appeared mostly random in both cell types at this early time point.

To determine whether infectious synapses formed as infected cells left the epithelium, we then analyzed the distribution of HIV-1 Gag in LC-TC conjugates harvested 60 hr after sheets from four donors were challenged with HIV-1_JR-CSF_. At this time, enrichment of Gag toward the LC-TC junction was apparent. To quantify the distribution of HIV-1 Gag in LC-TC conjugates, we assessed fluorescence in each of four segments of the cells. Of 66 Gag^+^ LC analyzed across the four donors, 51.5% displayed Gag signal primarily peripherally alongside the LC-TC contact zone, versus 7.6% toward the side farthest away from the TC (p < 0.0001) (Figure 6C). Of 175 Gag^+^ T cells analyzed across the four donors, 20.0% exhibited virus enrichment in the segment adjacent to the contact zone with the LC, versus 1.7% in the segment on the opposite side (p < 0.0001) (Figure 6D). These findings demonstrated that, during emigration of infected LC-TC conjugates from the vaginal epithelium, HIV concentrated toward the LC-TC junction in both cell types, supporting the formation of an infectious synapse.

## Discussion

HIV-1 attachment and penetration through the vaginal epithelium after sexual contact marks the initial step in establishing what now accounts for the majority of HIV-1 infections worldwide. Here, we showed by in situ investigations that both intraepithelial CD4^+^ T cells and LC in the human vagina were primary targets of HIV-1 infection. Heretofore, mucosal models have not permitted direct observation of these events within intact, viable vaginal outer epithelium. Our findings unequivocally demonstrated that upon first encounter, HIV-1 simultaneously entered LC and CD4^+^ T cells in the outer vaginal epithelium. Previous studies have suggested that HIV-1 sequentially binds to and infects dendritic cells (DC), which then transmit virus to adjacent T cells in the mucosal stroma and the local lymphatics (Geijtenbeek et al., 2000; Gurney et al., 2005). While this can still occur, our data showed that the initial infection was established in the outer epithelium where vaginal intraepithelial T cells bound and took up HIV-1 independent of LC, and likewise, intraepithelial LC rapidly internalized CCR5-tropic HIV-1.

The remarkable efficiency of HIV-1 entry into intraepithelial vaginal CD4^+^ T cells and LC appeared to be a manifestation of their expression of the major HIV-1 coreceptors, CD4 and CCR5 (Table 1). The majority (77%) of intraepithelial CD4^+^ T cells expressed CCR5, and LC commonly expressed CD4 (54%) and CCR5 (52%). Our findings were distinct from those previously reported, which failed to detect such high CCR5 expression on intraepithelial T cells and LC in the human vagina (Jameson et al., 2002; Rottman et al., 1997; Zhang et al., 1998). We believe that this discrepancy can be attributed principally to differences in the experimental procedures used for the analysis of the vaginal epithelium. Coreceptor expression of epithelial leukocytes was assessed here by immunofluorescent staining of live epithelial sheets or isolated cells shortly after surgical removal from the donor. In contrast to others, by refining our procedures, we surveyed the intact outer epithelium without treating with digestive enzymes or fixing, freezing, or embedding the tissue. Similarly, intraepithelial leukocytes were isolated mechanically rather than with proteolytic enzymes. These steps preserved cell viability and architecture, including surface molecule integrity, which likely increased the sensitivity and specificity of surface receptor detection. The importance of CCR5 expression in the two epithelial cell populations, LC and T cells, in their direct susceptibility to HIV-1 entry was confirmed here by strong (T cell) and partial (LC) inhibition of HIV-1 binding with CCR5 antibodies.

Although selective removal of LC from epithelial tissue sheets was not possible, we concluded that infection of intraepithelial CD4^+^ T cells did not require LC based on several lines of evidence reported here. First, the time (2–3 hr) from HIV challenge to subsequent detection of binding and entry in intraepithelial T cells was likely too short for virions to cycle sequentially from LC to T cells. Second, intraepithelial CD4^+^ T cells abundantly expressed CCR5, and this occurred independently of their contact with LC. Third, many single intraepithelial T cells exhibited viral binding and entry. Fourth, bound HIV-1 distributed randomly across the T cell surface at 2–3 hr after viral challenge. This was noted even among most T cells conjugated to LC in situ, indicating that HIV-1 accessed the T cells independently of LC. Fifth, the percentage of productively infected cells detected 3 days after viral challenge was not markedly higher in conjugated versus unconjugated T cells, suggesting that stable conjugation to LC was not required for productive infection of T cells.

Although HIV-1 efficiently entered both CD4^+^ T cells and LC, our findings indicated that the path of entry and fate of infection differed in the two cell populations (Table 1). Our data strongly suggested that HIV-1 infected intraepithelial CD4^+^ T cells in situ by fusion, as evidenced by the nearly complete dependency on CD4 and CCR5 coreceptors for HIV-1 entry as well as the detection of viral cores but not intact virions in the cytoplasm. Furthermore, productive HIV-1 infection was established in vaginal CD4^+^ T cells, indicated by viral protein synthesis in individual T cells within 2–3 days. By contrast, HIV-1 entry in vaginal LC occurred by endocytosis of intact virions. Remarkably, individual LC were heavily laden with virions when viewed 2 hr after challenge by electron microscopy, but productive HIV-1 infection in vaginal LC was not observed. We concede that our assays may have lacked the sensitivity to detect low-level viral protein synthesis in LC. Even so, if viral production occurred, it must have been relatively inefficient in contrast to the high capacity of LC to endocytose HIV-1.

Partial blocking of viral entry into vaginal LC by mannan in only two of three donors indicated that HIV-1 could utilize, but did not require, mannose binding C-type lectin receptors for endocytosis. This was consistent with a recent report demonstrating C-type lectin receptor-independent binding of HIV-1 to monocyte-derived DC (Gummuluru et al., 2003). Here, we also confirmed prior reports (Geijtenbeek et al., 2000; Jameson et al., 2002; Turville et al., 2002) that vaginal LC lack DC-SIGN and the mannose receptor (not shown), but do express langerin (CD207). Langerin or additional C-type lectin receptors may therefore be involved in endocytosis of HIV-1 into LC. Partial blocking of viral entry with CD4 and CCR5 antibodies, yet low or absent productive infection, raised the possibility that these classic fusion receptors participated in viral endocytosis. Alternatively, CD4 and CCR5 may have mediated fusion in LC, which we were unable to distinguish by fluorescence microscopy from endocytosed virions, whereas the block in viral replication occurred at a later stage (Hladik et al., 1999a). Taken together, these findings indicated that pathways of HIV-1 infection in vaginal LC were relatively more complex than in CD4^+^ T cells.

Our findings clearly demonstrated that both infected CD4^+^ T cells and LC were capable of migrating out of the vaginal epithelium, and thus both may be responsible for amplifying local infection and subsequently disseminating infection systemically. Our observation that intact virions remained in epithelial LC for at least 3 days supports the Trojan horse theory, which assumes that LC can harbor viable HIV over some period of time before passing it on to T cells (Geijtenbeek et al., 2000). Importantly, we observed enrichment of preserved or newly synthesized virus in the junction between vaginal LC and T cells emigrating out of the vaginal epithelium, indicative of an infectious synapse between the two cell types (Arrighi et al., 2004; Jolly and Sattentau, 2005; McDonald et al., 2003). Thus, it is likely that LC harboring HIV can spread infection to other target cells.

Our ex vivo organ culture system combined several advantages for investigating HIV-1 infection within the outer vaginal epithelium. In the epithelial sheets, HIV penetrated the tissue from both the apical and the basal side, which enhanced observation of the interactions between cells and virions. The sheets could be scanned in their full thickness by standard confocal microscopy, which permitted visualization of viral binding and entry within 24 hr in situ, before LC and T cells migrated from the epithelium into the culture medium. When productive infection was analyzed later in emigrated cells, prior separation of the epithelium from the underlying stroma ensured that only cells derived from the epithelial layer, and not stromal cells, were studied. Direct visualization of infection within individual cells by fluorescence microscopy, as employed in most experiments, also allowed exact identification of the specific cell type infected within the frequently occurring LC-TC conjugates.

In summary, we have provided definitive evidence that HIV-1 simultaneously entered CD4^+^ T cells and LC in the human vaginal epithelium. Direct productive infection of intraepithelial CD4^+^ T cells occurred very efficiently, reminiscent of the extraordinary HIV-1 susceptibility of CD4^+^ CCR5^+^ memory T cells inhabiting the gut mucosae (reviewed in Veazey and Lackner, 2005). Evidence that mAbs to CD4 and CCR5 effectively inhibited entry of CCR5-tropic HIV-1 into intraepithelial CD4^+^ T cells lends support to strategies employing CCR5-based microbicides for prevention of transmission in vivo, which have already shown to be quite effective in blocking SHIV infection in macaques (Lederman et al., 2004; Veazey et al., 2005). However, our findings also underscore the need for a topical microbicide to interfere with viral entry into LC as well, and this will not be achieved by blocking CCR5 alone. Consequently, this will require strategies both to counteract the rapid entry of HIV-1 into vaginal LC via multiple pathways and to capture the virus-laden LC prior to their exodus to the local lymphatics.

## Experimental Procedures

### Vaginal Epithelial Sheets

Tissues routinely discarded from vaginal repair surgeries in adult women were harvested, placed in ice-cooled phosphate-buffered saline (PBS), and transported to the laboratory within 1 hr of removal from the donor. Tissue harvesting and experimental procedures were approved by the Institutional Review Boards of the University of Washington and the Fred Hutchinson Cancer Research Center. Squamous epithelium was separated from vaginal stroma by either of three methods. For vacuum suction separation, the mucosa was pinned vaginal side up on cork glued to glass Eppendorf trays. The trays were placed on a heating plate set to 37°C, up to 14 custom-made suction cups were attached to the tissue, and culture medium (HEPES-buffered RPMI containing 10% fetal calf serum, 2 mM L-glutamine, 100 U/ml penicillin, and 100 μg/ml streptomycin) was added to the tray up to the level of the tissue stroma. Vacuum suction of ∼280 mmHg was applied for 2 hr, generating epithelial blisters 5 mm in diameter. Blister roofs were dissected off under a Zeiss KL1500 stereoscope, placed in cold PBS, and kept at 4°C until virus challenge.

For EDTA separation, the deep submucosa was removed with surgical scissors and the remaining mucosa was cut into 2 mm thin strips, placed in cold PBS containing 5 mM EDTA, and incubated at 4°C overnight under agitation. The epithelium was dissected off under the stereoscope, placed in Hanks buffered salt solution containing 5 mM calcium chloride for 1 hr on ice, washed in cold PBS, and used for virus challenge.

In three tissue donors, sections of epithelium were obtained by microdissection under a stereoscope, without either suction blistering or EDTA treatment. This procedure did not yield clean epithelial sheets and was used only for in situ immunofluorescence staining of CCR5. Confocal microscopy could be applied to scan limited areas of these native epithelia.

### Viruses

Molecular clones of HIV-1_JR-CSF_ and HIV-1 ΔEnv were fluorescent tagged as described (McDonald et al., 2002) by incorporation of a fusion protein of GFP and Vpr into the viral core. GFP-encoding, single round infection-competent, CCR5-tropic HIV-1 was generated by calcium phosphate-mediated transfection of HEK293T cells with the *Env* expression plasmid pCAGGS SF162 gp160 (Cheng-Mayer et al., 1997) and the *Env*-deleted provirus pLAI3ΔEnvGFP3 (Yamashita and Emerman, 2004). HIV-1_BaL_ was expanded in PHA-stimulated lymphoblasts. All viruses were concentrated 10- to 100-fold with Centricon Plus-80 100K centrifugal filter units (Millipore, Billerica, MA) and stored at −70°C. All virus preparations were assayed for infectivity in MAGI cells (Vodicka et al., 1997) or phytohemagglutinin (PHA)-activated lymphoblasts, and the Gag p24 concentration of the viral stocks was determined by an enzyme-linked HIV-1 p24 antigen capture assay (NCI-Frederick, Frederick, MD).

### Virus Inoculations

For viruses used, see Supplemental Experimental Procedures available online. For confocal fluorescence and electron microscopy of intact vaginal epithelium, epithelial sheets were cut into ∼1.5 × 1.5 mm pieces and placed into round-bottom 96-well plates filled with culture medium (50 μl/well). Some sheets were incubated at room temperature with either CD4 mAb (100 μg/ml RPA-T4), CCR5 mAb (100 μg/ml 2D7; both BD Biosciences, San Diego, CA), CD4 and CCR5 mAbs, or mannan (100 μg/ml; Sigma-Aldrich, St. Louis, MO) for 30 min before adding virus. Epithelial sheets were spinoculated at room temperature with viruses (200 ng/ml Gag p24 of GFP-tagged HIV-1_JR-CSF_ or HIV-1 ΔEnv; or 500 ng/ml Gag p24 of HIV-1_BaL_) for 2 hr at 1200 × g (O'Doherty et al., 2000), washed in staining buffer (SB; PBS containing 1% bovine serum albumin and 0.05% sodium azide), and either immunostained for confocal microscopy or fixed in Karnovsky's fixative for electron microscopy.

For detection of productive infection in LC and T cells emigrating from HIV-1-exposed vaginal epithelium, several uncut epithelial sheets (average size ∼5 × 5 mm) were placed in 6-well plates filled with culture medium (2 ml/well). Sheets were spinoculated with viruses (25 ng/ml Gag p24 of HIV-1_BaL_ for proviral PCR; 250 ng/ml Gag p24 of HIV-1_BaL_ for electron microscopy; 100 ng/ml Gag p24 of HIV-1_JR-CSF_; 25 ng/ml Gag p24 of GFP-encoding, single round infection-competent HIV-1 containing SF162 Env or ΔEnv) for 2 hr at 1200 × g (O'Doherty et al., 2000), washed in PBS six times, and cultured in culture medium for 60–72 hr at 37°C and 5% CO_2_. In HIV-1 PCR assays, we treated viruses with 30 U/ml of RNase-free DNase I (Boehringer Mannheim, Indianapolis, IN) before inoculation. In some sheets, 10 μM AZT (Sigma-Aldrich) was added during spinoculation and subsequent incubation. Emigrant cells from the epithelium were harvested from culture medium, and then either immunostained for fluorescence microscopy or flow cytometry or pelleted and fixed for electron microscopy.

### Immunostaining and Microscopy

Virus-challenged epithelial sheets were incubated in SB for 1 hr at RT with 10 μg/ml of one of the following antibodies: anti-CD4 (RPA-T4), anti-HLA-DR (G46-6; all BD Biosciences), or anti-CD1a (NA1/34; Dako, Carpinteria, CA). Sheets were washed in SB, incubated for 30 min with F(ab)2 of goat-anti-mouse conjugated to Alexa Fluor 568 (GAM AF568) (5 μg/ml; Molecular Probes, Eugene, OR), washed again, and fixed overnight in 4% paraformaldehyde. Nuclei were counterstained with TOPRO3 (1 μM; Molecular Probes), and the sheets were embedded in Mowiol 40-88 containing 2.5% w/v DABCO (Aldrich, Milwaukee, WI). Cellular staining was visualized with a Leica TCS SP spectral confocal microscope. Acquired image stacks were analyzed with Imaris software (Bitplane AG, Zurich, Switzerland). CCR5 and CD4 expression in situ was evaluated by staining in SB for 1 hr at RT with 10 μg/ml of fluorescein (FITC)-conjugated CCR5 mAb (2D7), CD4 mAb (RPA-T4) or isotype control, phycoerythrin (PE)-conjugated CD3 mAb (UCHT1), and allophycoerythrin (APC)-conjugated HLA-DR mAb (G46-6; all BD Biosciences) or CD1a mAb (NA1/34). In one tissue donor, anti-CCR5 PE was used in combination with anti-CD3 FITC. Native epithelia obtained by microdissection were stained with anti-CD1a (NA1/34), followed by GAM AF568 and anti-CCR5 FITC. Sheets were fixed, embedded, and viewed as above. Reflection-enhanced backscatter was used to visualize overall tissue structure.

Emigrated cells from virus-exposed sheets were incubated with 10 μg/ml of anti-HLA-DR, followed by GAM AF568 and anti-CD3 APC, each in SB for 30 min on ice. For intracellular HIV-1 Gag staining, cells were subsequently fixed, permeabilized, and incubated with anti-HIV-1 Gag FITC (KC57; Coulter, Fullerton, CA) according to the manufacturer's protocol. Cells were fixed in 4% paraformaldehyde and nuclei were counterstained for 10 min with 10 μg/ml diamidinophenylindole (DAPI; Molecular Probes), smeared on positively charged slides, embedded in Mowiol/DABCO, and viewed on a Deltavision SA3.1 wide-field deconvolution microscope (Applied Precision, Issaquah, WA). The algorithms used to quantify CCR5 and CD4 expression on LC in situ, binding and cytoplasmic entry of GFP-Vpr tagged HIV-1 in situ, and HIV-1 Gag and GFP expression in emigrated cells are described in the Supplemental Experimental Procedures available online.

Electron microscopy of epithelial sheets and emigrated cells was performed on a JEOL 1010 transmission electron microscope as previously described (Hladik et al., 1999a). Light microscopy was performed on paraffin-embedded, hematoxylin/eosin-stained, intact blisters and on toluidine blue-stained isolated epithelial sheets. The cell viability of isolated epithelial sheets was tested with calcein AM/ethidium homodimer-1 staining (Molecular Probes) as described in the manufacturer's protocol.

### Flow Cytometry

Receptor expression on intraepithelial T cells was performed by flow cytometry on cell suspensions obtained by dispersing vaginal epithelial sheets with the Biojector (Bioject Medical Technologies, Portland, OR) and filtering through a series of 200, 50, and 20 μm Filcons (BD Biosciences). Labeling of the cells with anti-CCR5 PE or isotype control antibody, anti-CD3 FITC, anti-CD4 APC (or vice versa) (all BD Biosciences), and propidium iodide (PI; Molecular Probes) was performed as described (Hladik et al., 1999b). For detection of HIV-1 Gag expression, flow cytometry was performed on emigrated cells from virus-exposed sheets. Cells were labeled with anti-CD3 APC and anti-HLA-DR PE, fixed, permeabilized, and labeled with anti-HIV-1 Gag FITC according to the manufacturer's protocol. Before the fixation step, the cells were stained with 7-amino-actinomycin D (7-AAD) (Sigma-Aldrich). Cells were acquired on a Calibur flow cytometer (BD Biosciences) and analyzed with CellQuest 3.3 (BD Biosciences) with gates set to isolate CD3^+^ or CD4^+^ T cells and to exclude PI^+^ or 7-AAD^+^ dead cells.

### PCR Assays for HIV-1 Proviral and Integrated Genomic DNA

Emigrated cells were harvested from virus-exposed epithelial sheets and DNA was isolated with the QiaAmp Blood Mini Kit (Qiagen, Valencia, CA). Proviral full-length *Env* DNA was amplified by nested PCR with primers and conditions previously described (Zheng and Daniels, 2001). Amplification of viral DNA integrated into the host cell genome, but not unintegrated viral input DNA, was performed with an *Alu*-LTR-based nested PCR assay as previously published (Brussel and Sonigo, 2003), except that 25 instead of 12 cycles were used in the first-round PCR. PCR products were visualized by electrophoresis in ethidium bromide-containing 4% agarose E-gel (Invitrogen, Carlsbad, CA). Parallel amplification of β-actin in each assay, with published primers and conditions (Zetterstrom et al., 2002), served to verify the presence of cellular input DNA.

### Statistics

To evaluate the significance of the difference in percent HIV-GFP^+^ cells between groups, the percentages of positive cells were averaged across all distinct, nonoverlapping confocal stacks within each donor and each experimental condition and compared by two-tailed one-sample t test. The two-tailed one-sample t test was also used to compare the percentage of emigrating cells expressing HIV-1 Gag. The significance of the difference in the number of cells with enrichment of HIV-1 Gag in one cell segment versus another was tested by two-tailed chi-square test.

## Figures and Tables

**Table 1:** HIV Receptor Profile and Initial Infection Events in the Human Vaginal Epithelium

	CD4^+^ T Cells	Langerhans Cells
Intraepithelial Cells		

HIV-1 Receptor Expression		
CD4	100%∗	54%
CCR5	77%	52%
DC-SIGN	(−)∗∗	(−)
Mannose receptor	(−)	(−)
Langerin	(−)	(+)
HIV-1 Binding/Entry		
Cytoplasmic entry	(++)	(++)
Endocytosis	(−)	(++)
Anti-CD4 Ab inhibition	(++)	(+)
Anti-CCR5 Ab inhibition	(++)	(+)
Mannan inhibition	(−)	(+/−)
Cellular HIV-1 distribution	random	random

Emigrant Cells		

Intracellular Gag protein	(+)	(+)
Intact cytoplasmic virions	(−)	(+)
Productive infection	(+)	(−)∗∗∗
Cellular HIV-1 distribution	LC-T cell junction	LC-T cell junction

∗ 27% of intraepithelial T cells were CD4^+^.

∗∗ (−) not detected; (+) detected; (++) strongly detected.

∗∗∗ Very low-level productive infection of LC could not be excluded by our experimental procedures.

## Supplemental Data

Document S1. Three Figures and Experimental Procedures

Movie S1. Three-Dimensional Reconstruction from a Confocal Image Stack of an Intraepithelial CD4+ T Cell by Imaris Software The cell was virtually clipped at its widest circumference so that the green virions located inside the cytoplasm, between the red cell membrane and the blue nucleus, can be clearly identified. Areas where HIV-1 penetrates the cell membrane, signified by CD4 and HIV-1 colocalization, are shown in white color. The nucleus is rendered as an isosurface and virions appear to enter it at one location.

## References

[bib1] Arrighi J.F., Pion M., Garcia E., Escola J.M., van Kooyk Y., Geijtenbeek T.B., Piguet V. (2004). DC-SIGN-mediated infectious synapse formation enhances X4 HIV-1 transmission from dendritic cells to T cells. J. Exp. Med..

[bib2] Brussel A., Sonigo P. (2003). Analysis of early human immunodeficiency virus type 1 DNA synthesis by use of a new sensitive assay for quantifying integrated provirus. J. Virol..

[bib3] Cheng-Mayer C., Liu R., Landau N.R., Stamatatos L. (1997). Macrophage tropism of human immunodeficiency virus type 1 and utilization of the CC-CKR5 coreceptor. J. Virol..

[bib4] Collins K.B., Patterson B.K., Naus G.J., Landers D.V., Gupta P. (2000). Development of an in vitro organ culture model to study transmission of HIV-1 in the female genital tract. Nat. Med..

[bib5] Dimitrov D.S., Broder C.C., Berger E.A., Blumenthal R. (1993). Calcium ions are required for cell fusion mediated by the CD4-human immunodeficiency virus type 1 envelope glycoprotein interaction. J. Virol..

[bib6] Geijtenbeek T.B., Kwon D.S., Torensma R., van Vliet S.J., van Duijnhoven G.C., Middel J., Cornelissen I.L., Nottet H.S., KewelRamani V.N., Littman D.R. (2000). DC-SIGN, a dendritic cell-specific HIV-1-binding protein that enhances trans-infection of T cells. Cell.

[bib7] Greenhead P., Hayes P., Watts P.S., Laing K.G., Griffin G.E., Shattock R.J. (2000). Parameters of human immunodeficiency virus infection of human cervical tissue and inhibition by vaginal virucides. J. Virol..

[bib8] Gummuluru S., Rogel M., Stamatatos L., Emerman M. (2003). Binding of human immunodeficiency virus type 1 to immature dendritic cells can occur independently of DC-SIGN and mannose binding C-type lectin receptors via a cholesterol-dependent pathway. J. Virol..

[bib9] Gupta P., Collins K.B., Ratner D., Watkins S., Naus G.J., Landers D.V., Patterson B.K. (2002). Memory CD4(+) T cells are the earliest detectable human immunodeficiency virus type 1 (HIV-1)-infected cells in the female genital mucosal tissue during HIV-1 transmission in an organ culture system. J. Virol..

[bib10] Gurney K.B., Elliott J., Nassanian H., Song C., Soilleux E., McGowan I., Anton P.A., Lee B. (2005). Binding and transfer of human immunodeficiency virus by DC-SIGN+ cells in human rectal mucosa. J. Virol..

[bib11] Hladik F., Lentz G., Akridge R.E., Peterson G., Kelley H., McElroy A., McElrath M.J. (1999). Dendritic cell-T-cell interactions support coreceptor-independent human immunodeficiency virus type 1 transmission in the human genital tract. J. Virol..

[bib12] Hladik F., Lentz G., Delpit E., McElroy A., McElrath M.J. (1999). Coexpression of CCR5 and IL-2 in human genital but not blood T cells: implications for the ontogeny of the CCR5+ Th1 phenotype. J. Immunol..

[bib13] Hu J., Gardner M.B., Miller C.J. (2000). Simian immunodeficiency virus rapidly penetrates the cervicovaginal mucosa after intravaginal inoculation and infects intraepithelial dendritic cells. J. Virol..

[bib14] Hu Q., Frank I., Williams V., Santos J.J., Watts P., Griffin G.E., Moore J.P., Pope M., Shattock R.J. (2004). Blockade of attachment and fusion receptors inhibits HIV-1 infection of human cervical tissue. J. Exp. Med..

[bib15] Jameson B., Baribaud F., Pohlmann S., Ghavimi D., Mortari F., Doms R.W., Iwasaki A. (2002). Expression of DC-SIGN by dendritic cells of intestinal and genital mucosae in humans and rhesus macaques. J. Virol..

[bib16] Jolly C., Sattentau Q.J. (2005). Human immunodeficiency virus type 1 virological synapse formation in T cells requires lipid raft integrity. J. Virol..

[bib17] Kawamura T., Cohen S.S., Borris D.L., Aquilino E.A., Glushakova S., Margolis L.B., Orenstein J.M., Offord R.E., Neurath A.R., Blauvelt A. (2000). Candidate microbicides block HIV-1 infection of human immature Langerhans cells within epithelial tissue explants. J. Exp. Med..

[bib18] Klimstra W.B., Nangle E.M., Smith M.S., Yurochko A.D., Ryman K.D. (2003). DC-SIGN and L-SIGN can act as attachment receptors for alphaviruses and distinguish between mosquito cell- and mammalian cell-derived viruses. J. Virol..

[bib19] Lederman M.M., Veazey R.S., Offord R., Mosier D.E., Dufour J., Mefford M., Piatak M., Lifson J.D., Salkowitz J.R., Rodriguez B. (2004). Prevention of vaginal SHIV transmission in rhesus macaques through inhibition of CCR5. Science.

[bib20] Maher D., Wu X., Schacker T., Horbul J., Southern P. (2005). HIV binding, penetration, and primary infection in human cervicovaginal tissue. Proc. Natl. Acad. Sci. USA.

[bib21] McDonald D., Vodicka M.A., Lucero G., Svitkina T.M., Borisy G.G., Emerman M., Hope T.J. (2002). Visualization of the intracellular behavior of HIV in living cells. J. Cell Biol..

[bib22] McDonald D., Wu L., Bohks S.M., Kewal V.N., Unutmaz D., Hope T.J. (2003). Recruitment of HIV and its receptors to dendritic cell-T cell junctions. Science.

[bib23] O'Doherty U., Swiggard W.J., Malim M.H. (2000). Human immunodeficiency virus type 1 spinoculation enhances infection through virus binding. J. Virol..

[bib24] Rottman J.B., Ganley K.P., Williams K., Wu L., Mackay C.R., Ringler D.J. (1997). Cellular localization of the chemokine receptor CCR5. Correlation to cellular targets of HIV-1 infection. Am. J. Pathol..

[bib25] Spira A.I., Marx P.A., Patterson B.K., Mahoney J., Koup R.A., Wolinsky S.M., Ho D.D. (1996). Cellular targets of infection and route of viral dissemination after an intravaginal inoculation of simian immunodeficiency virus into rhesus macaques. J. Exp. Med..

[bib26] Turville S.G., Cameron P.U., Handley A., Lin G., Pohlmann S., Doms R.W., Cunningham A.L. (2002). Diversity of receptors binding HIV on dendritic cell subsets. Nat. Immunol..

[bib27] Veazey R.S., Lackner A.A. (2005). HIV swiftly guts the immune system. Nat. Med..

[bib28] Veazey R.S., Marx P.A., Lackner A.A. (2003). Vaginal CD4+ T cells express high levels of CCR5 and are rapidly depleted in simian immunodeficiency virus infection. J. Infect. Dis..

[bib29] Veazey R.S., Klasse P.J., Schader S.M., Hu Q., Ketas T.J., Lu M., Marx P.A., Dufour J., Colonno R.J., Shattock R.J. (2005). Protection of macaques from vaginal SHIV challenge by vaginally delivered inhibitors of virus-cell fusion. Nature.

[bib30] Vodicka M.A., Goh W.C., Wu L.I., Rogel M.E., Bartz S.R., Schweickart V.L., Raport C.J., Emerman M. (1997). Indicator cell lines for detection of primary strains of human and simian immunodeficiency viruses. Virology.

[bib31] Yamashita M., Emerman M. (2004). Capsid is a dominant determinant of retrovirus infectivity in nondividing cells. J. Virol..

[bib32] Zetterstrom C.K., Bergman T., Rynnel-Dagoo B., Erlandsson Harris H., Soder O., Andersson U., Boman H.G. (2002). High mobility group box chromosomal protein 1 (HMGB1) is an antibacterial factor produced by the human adenoid. Pediatr. Res..

[bib33] Zhang L., He T., Talal A., Wang G., Frankel S.S., Ho D.D. (1998). In vivo distribution of the human immunodeficiency virus/simian immunodeficiency virus coreceptors: CXCR4, CCR3, and CCR5. J. Virol..

[bib34] Zhang Z., Schuler T., Zupancic M., Wietgrefe S., Staskus K.A., Reimann K.A., Reinhart T.A., Rogan M., Cavert W., Miller C.J. (1999). Sexual transmission and propagation of SIV and HIV in resting and activated CD4+ T cells. Science.

[bib35] Zheng N.N., Daniels R.S. (2001). Maintenance of glycoprotein-determined phenotype in an HIV type 1 (pNL43) env gene-cassetting system. AIDS Res. Hum. Retroviruses.

