# Supplementary material for: Initial Events in Establishing Vaginal Entry and Infection by Human Immunodeficiency Virus Type-1
Source: Immunity. 2007 Feb 23;26(2):257–70. doi: 10.1016/j.immuni.2007.01.007 (PMC1885958; doi:10.1016/j.immuni.2007.01.007)
Supplement: Document S1. Three Figures and Experimental Procedures [file mmc1.pdf]

**Immunity, Volume *26***

# Supplemental Data

# Initial Events in Establishing Vaginal Entry and Infection by Human Immunodeficiency Virus Type-1

**Florian Hladik, Polachai Sakchalathorn, Lamar Ballweber, Gretchen Lentz, Michael Fialkow, David Eschenbach, and M. Juliana McElrath**

## Supplemental Experimental Procedures

### Algorithms applied for quantitation of receptor- or HIV-positive cells.

(1) CCR5 and CD4 expression on Langerhans cells *in situ*.
To determine the percentage of $CCR5^+$ and $CD4^+$ LC *in situ*, we acquired 2-5 single 200×200 μm planes per stained epithelial sheet and counted all HLA-DR- or CD1a-positive LC, as well as the number of LC expressing either CCR5 or CD4, using softWoRx (Applied Precision). A Langerhans cell was designated receptor-positive when its green fluorescence was >1.5× the fluorescence in any of eight 20×20 μm fields immediately adjacent to the cell (fields containing additional positive cells were excluded).

(2) Binding of GFP-tagged HIV-1 *in situ*.
To enumerate $CD4^+$ T cells and $CD1a^+$ LC *in situ* binding GFP-tagged HIV-1, we acquired 3-5 distinct confocal stacks per epithelial sheet at 20× and counted HIV-positive and HIV-negative cells within the resulting 200×200×40 μm cubes using MetaMorph 6.2 (Molecular Devices, Downingtown, PA). A $CD4^+$ T cell was designated HIV-positive when at least one quarter of its circumference at any single-plane z-section displayed binding of green virions. A $CD1a^+$ LC was designated HIV-positive when its green fluorescence was >3× the fluorescence in any of eight 20×20 μm fields immediately adjacent to the cell (fields containing additional positive cells were excluded).

(3) Cytoplasmic entry of GFP-tagged HIV-1 *in situ*.
Viral entry into intraepithelial $CD4^+$ T cells was determined after acquisition of randomly selected $GFP^+$ $CD4^+$ T cells at 60× and deconvolution by Autodeblur (Autoquant, Watervliet, NY). Entry of HIV into the cytoplasm was judged to have occurred if a GFP signal was detected inside the cell and immediately adjacent to a surface area displaying colocalization of GFP and CD4. Entry was assessed only at the widest circumference of a cell, decreasing the likelihood of non-specific fluorescence projections. Viral entry into $CD1a^+$ LC was easily discerned without deconvolution and formal colocalization studies.

(4) Gag p55/p24 detection in emigrated cells.
LC-T cell conjugates were identified on the slide by the bright red HLA-DR fluorescence of the LC, and a 0.2 μm optical section through the interior of the cells was acquired under identical microscope settings. Within a conjugate, each T cell and LC was assessed for its maximum fluorescence intensity (MAX) using softWoRx software (Applied Precision). The MAX of each individual cell within each conjugate in the no-virus control sample was divided by the minimum fluorescence intensity (MIN) given for each respective conjugate by softWoRx. The MAX / MIN values for all T cells, and all LC, in the no-virus control sample were averaged separately, and two standard deviations were added to yield a single MAX / MIN control value (CV) for T cells, and a single CV for LC. To calculate the positive-negative cutoff (PNC) for each individual T cell or LC, the respective CV was multiplied by the specific MIN of that cell. This procedure ensured an individualized cutoff determination for each cell that takes into account variations of background staining between different cells. To obtain the Gag-specific fluorescence level (GF) for each individual conjugated cell, the PNC was subtracted from the MAX of each cell. Thus, for each individual

cell, GF = MAX – (CV × MIN).

(5) Pseudotype GFP expression in emigrated cells.
For microscopic analysis of cellular GFP expression following infection with the HIV-1 SF162 Env pseudotype, we chose a computerized method allowing the evaluation of large numbers of T cells and LC. Glass slides with emigrated cells were scanned by preset automatic stage movements on the Deltavision microscope. Adjacent 20× fields covering the complete area of the cell smear were automatically acquired in the blue, green, red and far red light emission spectrum. Computerized identification and marking of $DAPI^+$ $CD3^+$ $HLA\text{-}DR^-$ T cells and $DAPI^+$ $CD3^-$ $HLA\text{-}DR^+$ LC were performed using ImageJ 1.37 open source software. Optimal detection thresholds for DAPI, HLA-DR GAM AF 568 and CD3 APC were determined in preliminary tests. The accuracy of cell type identification was visually checked, excluding from further analysis falsely identified or uninterpretable events such as epithelial cells or large cell clusters. The maximum GFP fluorescence of each cell was then plotted separately for T cells and LC, and the percentage of GFP-positive cells within each cell subset was calculated using an arbitrary cut-off set by comparison to the ΔEnv control sample.

**Supplemental Reference**

Bieber, T., Jurgens, M., Wollenberg, A., Sander, E., Hanau, D., and de la Salle, H. (1995). Characterization of the protein tyrosine phosphatase CD45 on human epidermal Langerhans cells. Eur J Immunol 25, 317-321.

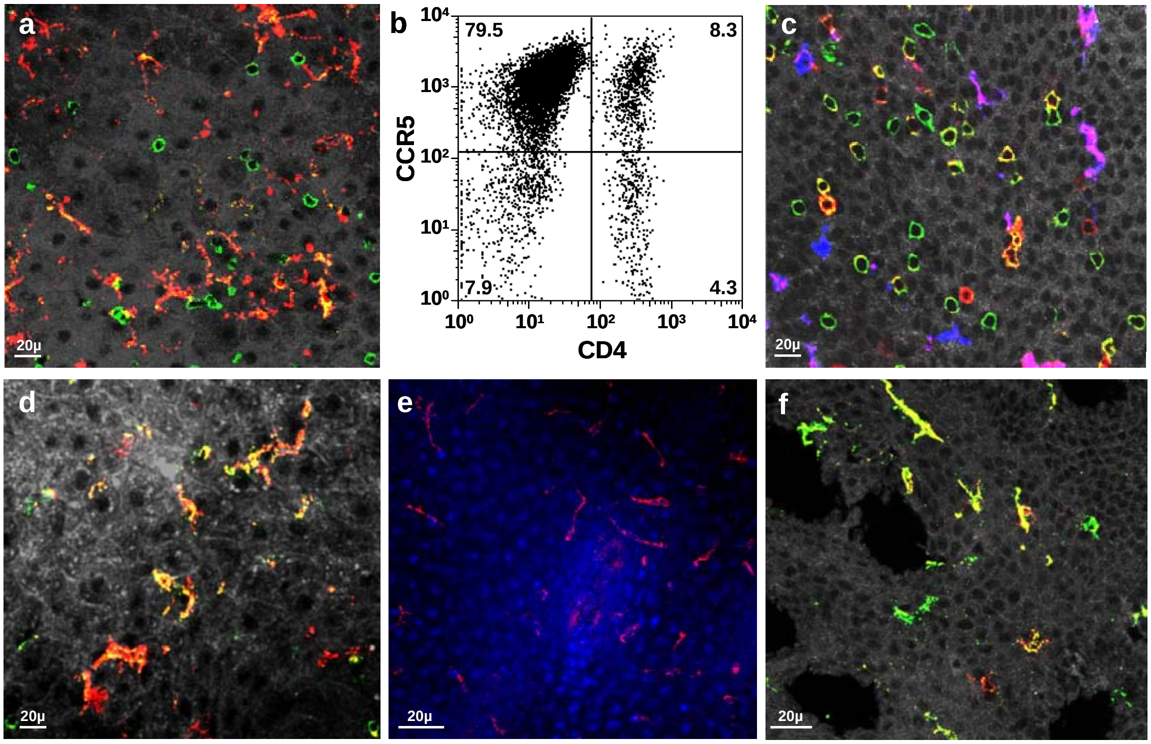


**Figure S1. CD4, CCR5 and Langerin Expression on Intraepithelial Vaginal T Cells and Langerhans Cells**

**(a)** *In situ* CCR5 fluorescence staining of vaginal epithelium microdissected from the underlying stroma without suction blistering or EDTA treatment. Representative photomicrograph of three donor experiments. Expression of CCR5 is shown in green and CD1a in red. Yellow signifies co-expression of CCR5 and CD1a. Circular green staining is typical of CCR5 expressing T cells. **(b-d)** CD4 and CCR5 expression in EDTA-separated sheets. **(b)** Representative flow cytometry plot of mechanically isolated, CD3-gated, intraepithelial T cells in one of three tissue donors. Propidium iodide-positive dead cells were excluded from analysis. Quadrants were set in reference to staining with isotype control antibodies. The percentages of cells in each quadrant that express CCR5 and/or CD4 are indicated. **(c)** *In situ* fluorescence staining for CCR5, representative of four donors. The CD3 label is shown in green, CCR5 in red and CD1a in blue. Yellow signifies co-expression of CD3 and CCR5. Pink signifies co-expression of CD1a and CCR5. **(d)** *In situ* fluorescence staining for CD4, representative of three donors. The CD4 label is shown in green, the HLA-DR label in red. HLA-DR labels mostly dendritic-shaped LC. Yellow signifies co-expression of CD4 and HLA-DR. **(e-f)** Langerin expression by vaginal LC**.** **(e)** Vaginal epithelial sheets were incubated with anti-langerin, followed by goat-anti-mouse Alexa Fluor 568. The blue nuclear counterstain is Topro-3. **(f)** Vaginal epithelial sheets were incubated with anti-langerin, followed by goat-anti-mouse Alexa Fluor 568, and finally by anti-CD1a FITC. Green signifies CD1a expression, red langerin and yellow co-expression of both molecules. In a, c, d and f, reflection-enhanced backscatter was used to visualize overall tissue structure (grey).

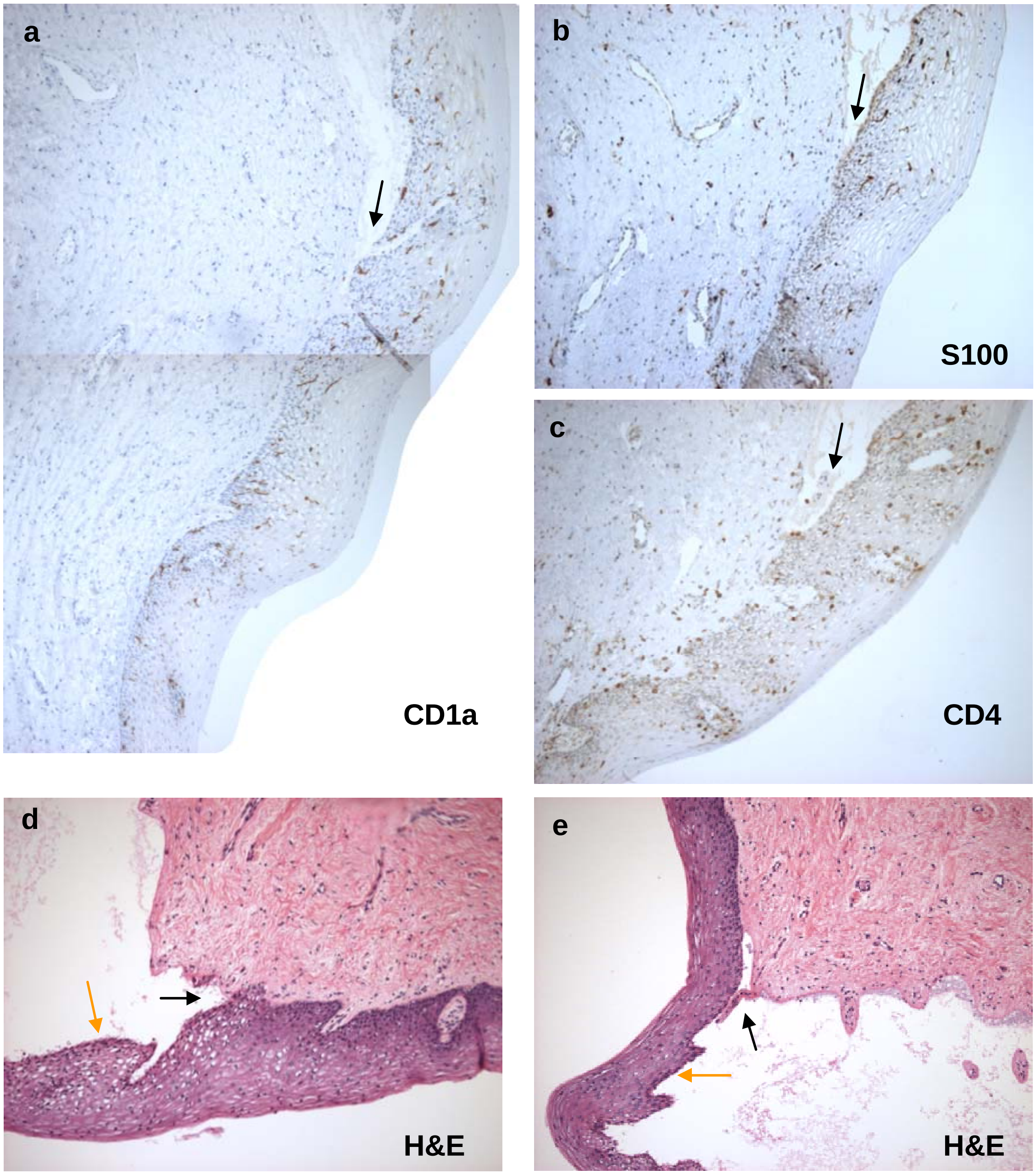


**Figure S2. Distribution of LC, Dendritic Cells, and CD4$^+$ T Cells in Blister Roof versus Adjacent Unseparated Vaginal Epithelium**
**(a)** Immunohistochemistry for CD1a reveals CD1a$^+$ LC only within the epithelium. No CD1a$^+$ cells are present in the vaginal stroma. The distribution of LC within separated versus adjacent unseparated epithelium appears to be similar. Black arrows point to the separation zone where the blister forms. **(b)** The pan-mDC marker S100 is expressed by LC within the vaginal epithelium as well as by DC in the stroma. The distribution of DC within separated versus adjacent unseparated epithelium appears to be similar. **(c)** Like DC, the distribution of CD4$^+$ T cells appears similar between separated versus adjacent unseparated epithelium. CD4$^+$ T cells are found both within the epithelium and the stroma. **(d-e)** H&E stain of the same blister as in (a-c). Both separation zones present on the histological cross-section are shown. The basal layer of the separated epithelium is intact on both sides of the blister (orange arrows).

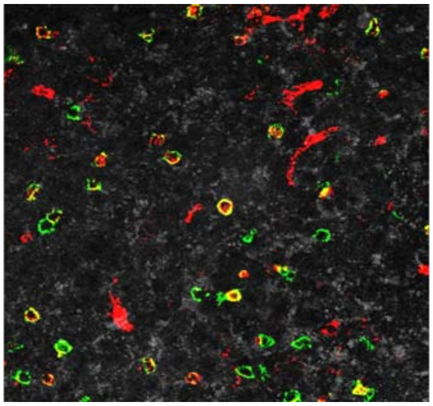

**Figure S3. CD45RO Expression by Vaginal Intraepithelial T Cells**
Staining of vaginal epithelium was performed with anti-CD45RO, followed by GAM AF568, and finally anti-CD3 FITC. Many $CD3^+$ T cells (green) express CD45RO (yellow signifies CD3/CD45RO co-expression). Some LC also express CD45RO (red), which is in line with other reports (Bieber et al., 1995).
